# Functional Characterization of the Internal Symmetry of MRAP2 Antiparallel Homodimer

**DOI:** 10.3389/fendo.2021.750797

**Published:** 2021-10-25

**Authors:** Meng Wang, Linyu Pi, Xiaowei Lei, Lei Li, Jing Xu, Zhe Kuang, Cong Zhang, Liang Li, Chao Zhang

**Affiliations:** ^1^ Department of Plastic and Reconstructive Surgery, Shanghai Institute of Precision Medicine, Shanghai Ninth People’s Hospital, Key Laboratory of Cell Differentiation and Apoptosis of Chinese Ministry of Education, Shanghai Jiao Tong University School of Medicine, Shanghai, China; ^2^ Translational Medical Center for Stem Cell Therapy and Institute for Regenerative Medicine, Shanghai East Hospital, Shanghai Key Laboratory of Signaling and Disease Research, School of Life Sciences and Technology, Tongji University, Shanghai, China; ^3^ Department of Thyroid and Breast Surgery, ZiBo Central Hospital Affiliated of Binzhou Medical University, Zibo, China

**Keywords:** MRAP2, GPCR, dimerization, internal symmetry, MC4R

## Abstract

The melanocortin receptors are defined as a series of vital pharmaceutical targets to regulate neuronal appetite and maintain controllable body weight for mammals and teleosts. Melanocortin receptor accessory protein 2 (MRAP2) functions as an essential accessory player that modulates the surface translocation and binding to a variety of endogenous or synthetic hormones of central melanocortin-4 receptor (MC4R) signaling. MRAP2 is a single-transmembrane protein and could form a functional symmetric antiparallel homodimer topology. Here, we inverted the N-terminal, transmembrane, and C-terminal domains and generated six distinct conformational variants of the mouse MRAP2 to explore the functional orientations and the internal symmetry of MRAP2 dimers. These remolded MRAP2 mutants showed proper assembly of the antiparallel homodimer and binding to the MC4R, but slightly altered the regulatory profile on the surface expression and the ligand-stimulated cAMP cascades of MC4R. This study elucidated the importance of the orientation of each domain of the single-transmembrane protein and revealed the pharmacological properties of the internal symmetry of the antiparallel homodimer for MRAP2.

## Introduction

Melanocortins consist of four pro-opiomelanocortin (POMC)-derived natural agonists (α-, β-, and γ-MSH and ACTH) and two antagonists (Agouti and AgRP) through tissue-specific posttranslational processing and modification (1). The biological activity and physiological roles of melanocortins are mediated by five melanocortin receptors (MCRs) in mammals (MC1R–MC5R) (2). In the skin and hair follicles, alpha melanocyte-stimulating hormone (α-MSH)-simulated MC1R signaling results in melanin production producing black skin or hair (3). MC2R is required for adrenocortical steroidogenesis in the adrenal gland, and melanocortin-3 receptor (MC3R) and MC4R are essential for appetite control and energy homeostasis in the central nervous system.Mutations of MC4R have been reported as the most prevalent forms of monogenic obesity in humans, and the physiological roles of MC4R in regulating energy balance are well known and widely reported (4, 5). Remarkably, MC4R is defined as an ideal pharmaceutical target for screening allosteric modulators in order to treat metabolic disorders or maintain controllable body weight for professional athletes such as those in weightlifting, boxing, judo, and artistic gymnastics (6–8). MC5R plays an important role in the exocrine secretion of the skin (9, 10).

Melanocortin receptor accessory proteins (MRAPs) are small single-transmembrane proteins that regulate the intracellular cyclic adenosine monophosphate (cAMP) signaling and cell surface translocation of MCRs (11). By studying the familial glucocorticoid deficiency (FGD), Noon and his colleagues first found that the trafficking of MC2R to the cell surface required the melanocortin receptor accessory protein 2 (MRAP2) in the adrenal glands (12). Subsequently, MRAP2 was identified as a paralog of MRAP and exhibited universal interaction with all five MCRs. MRAP2 was highly expressed in the paraventricular nucleus (PVN) of the hypothalamus and served as the key player in the central regulation of energy expenditure and appetite (13). *In vivo* studies on zebrafish and mice also supported the notion that MRAP2 was an allosteric modulator and pharmacological partner for the regulation of MC4R signaling (14, 15).

MRAP1 and MRAP2 both contain a glycosylation site in the N-terminal and assemble an antiparellel dual topology on the plasma membrance, indicating that MRAPs simultaneously insert into the membrane as Ncyt/Cexo (N-terminal intracellular/C-terminal extracellular) and Nexo/Ccyt (N-terminal intracellular/C-terminal extracellular) orientations. Inversely oriented MRAP1 and MRAP2 could form symmetic antiparallel homodimers and heterodimers (16). As a functional protein complex, the antiparellel dimeric topology of MRAPs exhibited an indispensable extrinsic symmetry. However, due to the lack of a crystal structure of MRAP2, the intrinsic symmetry of MRAP2 dimers remains unclear. We speculate that alterations in the orientation of the internal domains of MRAPs could potentially optimize the regulatory function on the melancortin receptors without changing the extrinsic symmetric topology. Investigation of the internal symmetry of antiparellel MRAP2 dimers has become of vital importance. In this study, we artifically constructed six variant conformations of mouse MRAP2 (mMRAP2) as follows: transmembrane region (TM) inversion (I), N-terminal inversion (II), C-terminal inversion (III), N- and C-terminal inversion (IV), N- and C-terminal transposition (V), and replacement of the C-terminal by the inverted N-terminal (VI). Our results demonstrated that the alterations of the internal symmetry did not affect the proper formation of the antiparallel homodimer or the binding to mouse melanocortin receptor 4 (mMC4R). However, distinct variants showed variable pharmacological regulations and effects on the membrane transport of mMC4R. Among them, the N-terminal inverted variant exhibited the greatest impact on the constitutive activity of mMC4R. Overall, our study confirmed the symmetric topology of the MRAP2 homodimer and elucidated the importance of the orientation of each interior domain for the functional modulation of MC4R signaling.

## Results

### Interactions of MRAP2 Protein Variants With MC4R

**Figure 1:**
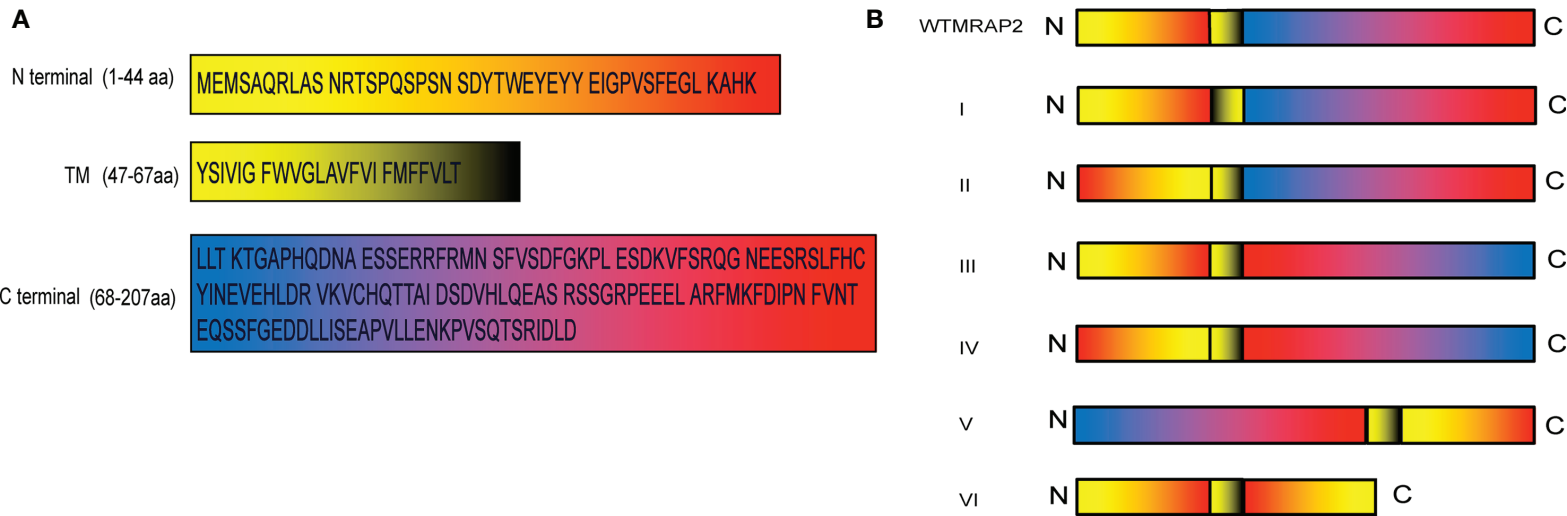
**(A)** Sequences of mouse melanocortin receptor accessory protein 2 (mMRAP2). The different colors of the sequences in the *black box* indicate the N-terminal, transmembrane (TM), and C-terminal domains. **(B)** Schematic representation of the six MRAP2 variants. From *top* to *bottom*: wild-type MRAP2 (wtMRAP2), I (TM inversion), II (N-terminal inversion), III (C-terminal inversion), IV (N- and C-terminal inversion), V (N- and C-terminal transposition), and VI (replacement of C-terminal by the inverted N-terminal).

**Figure 2:**
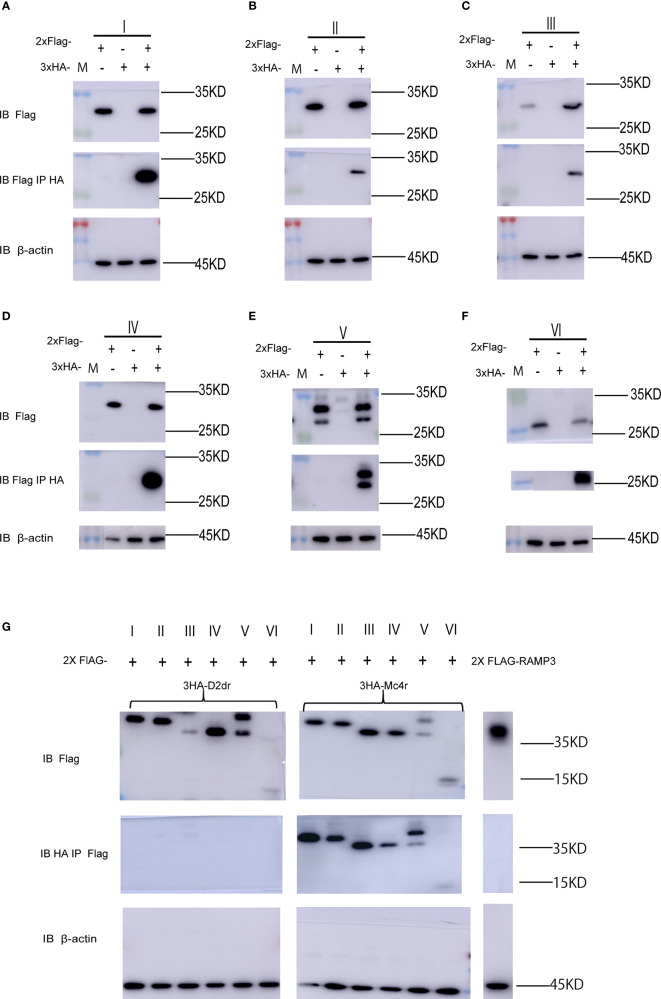
Melanocortin receptor accessory protein 2 (MRAP2) variants form proper homodimers and interact with melanocortin-4 receptor (MC4R). **(A–F)** Co-immunoprecipitation (IP) of the 2FLAG-tagged and 3HA-tagged MRAP2 variants I, II, III, IV, V, and VI. The order of transfection in each group is from *left* to *right*: 2FLAG-tagged variant transfected only, 3HA-tagged variant transfected only, and 2FLAG-tagged and 3HA-tagged variants co-transfected. **(G)** Immunoprecipitation and Western blot detection of 3HA-D2dr, 3HA-MC4R, and 2Flag-MRAP2 variants or RAMP3. MRAP2 variants and RAMP3 were detected using mouse anti-Flag antibody. *IB*, immunoblotting, beads only, no antibody was used for the IP.

As shown in **Figure 1A**, the full-length mMRAP2 has a total of 207 amino acids, including that of the N-terminal domain (amino acids 1–46), the TM region (amino acids 47–67), and the C-terminal domain (amino acids 68–207). We artifically constructed six variants of mMRAP2 as follows: TM invertion (I), N-terminal invertion (II), C-terminal invertion(III), N- and C-terminal invertion (IV), N- and C-terminal transposition (V), and replacement of the C-terminal by the inverted N-terminus (VI) (**Figure 1B**). Next, we transfected HEK293T cells with hemagglutinin (HA)- and FLAG-tagged mMRAP2 at a ratio of 1:1 and performed co-IP analysis. Our results demonstrated the proper homodimerization of these variants, suggesting a robust interaction between these variant monomers (**Figures 2A–F**).

Subsequently, we co-transfected the mMC4R and mMRAP2 plasmids into HEK293 cells to examine the interaction of the MRAP2 variants with their G-protein-coupled receptor (GPCR) targets. Co-immunoprecipitation (co-IP) assay exhibited the proper interaction of all six MRAP2 variants with MC4R *in vitro*, suggesting that the alteration of the interior orientation of each domain did not affect the interaction between these mutant mMRAP2 and mMC4R (**Figure 2G**). RAMP3 (another single-transmembrane accessory protein) was utilized as a control group and showed no interplay with MC4R (**Figure 2G**). Meanwhile, co-IP of the dopamine receptor D2 with MRAP2 excluded nonspecific binding and immunoprecipitation (IP) artifacts, and β-actin was recruited as an internal reference for all co-IP analyses.

### Pharmacological Effect of MRAP2 Variants on α-MSH-Induced MC4R Activity

**Figure 3:**
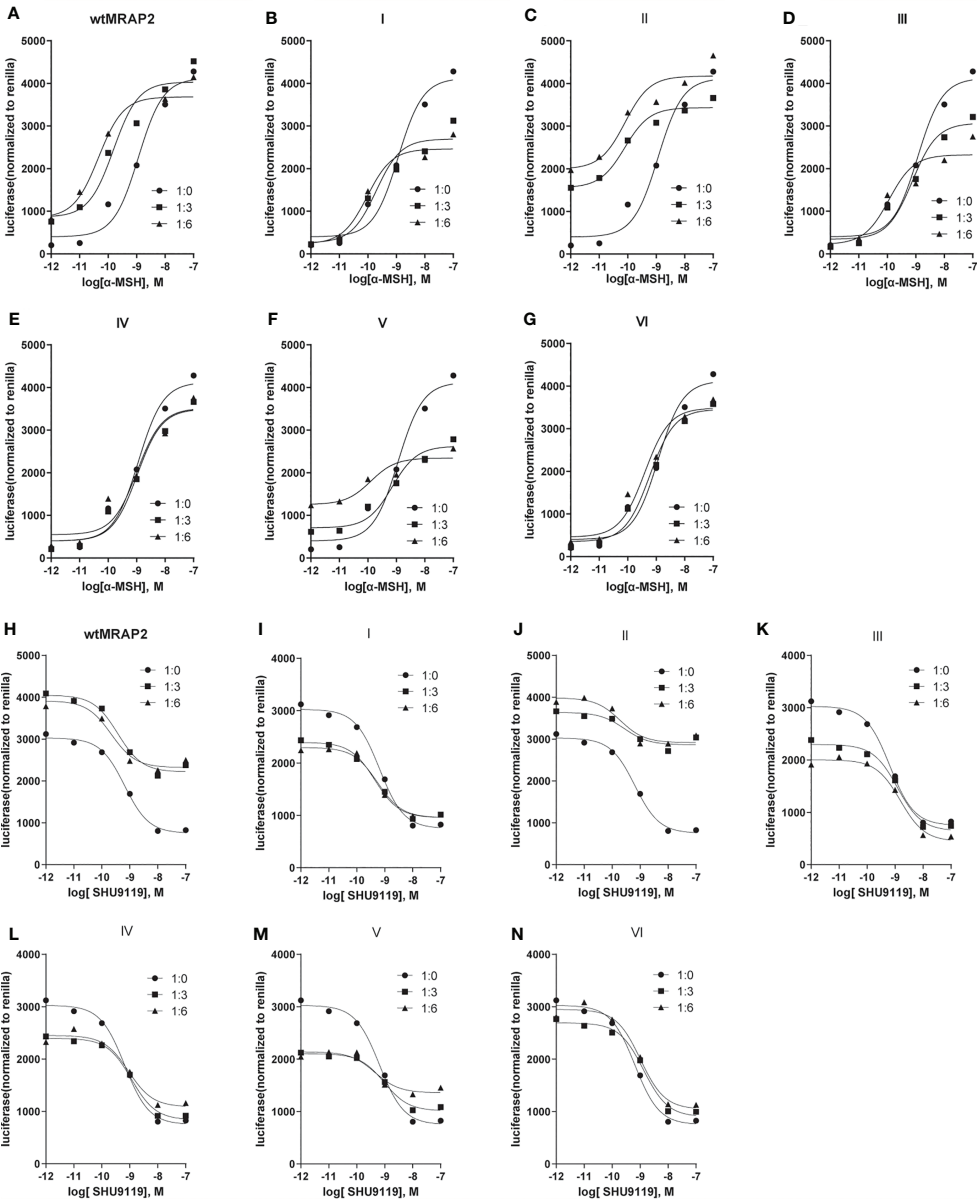
Pharmacological modulation of melanocortin-4 receptor (MC4R) signaling by melanocortin receptor accessory protein 2 (MRAP2) variants. **(A–G)** cAMP response to increasing concentrations of alpha melanocyte-stimulating hormone (α-MSH; 10^−7^–10^−12^ M) in HEK293T cells transfected with MC4R and an empty vector or in the presence of different ratios of MRAP2 or the MRAP2 variants (1:0, 1:3, and 1:6). Relative luminescence intensity of pCre-Luc represents the normalized pCre-Luc units to pRL-TK units (transfected internal control). **(H–N)** Binding competition of the agonist (α-MSH) and antagonist (SHU9119) of MC4R modulated by the MRAP2 variants. The relative luminescence intensity of pCre-Luc represents the normalized pCre-Luc units to pRL-TK units (transfected internal control). Each *data point* represents the mean ± SEM of three replicates (*N* = 3).

To investigate the pharmacological regulation of each MRAP2 variant on MC4R signaling, CRE-luciferase reporter assay was performed to detect the cAMP level stimulated by α-MSH in the presence of wild-type MRAP2 (wtMRAP2) and the six MRAP2 variants. It has been well documented that MRAP2 formed dimers or even higher-order oligomers to further interact with individual receptors, and the high-level functional unit may underlie the concentration-dependent effect of the regulatory action of MRAP2 on MC4R (16–19). Therefore, MC4R was co-transfected with wtMRAP2 or each variant into HEK293T cells at ratios of 1:0, 1:3, and 1:6. As shown in **Figure 3A**, MRAP2 significantly potentiated the EC_50_ of MC4R activity in a dose-dependent manner in subnanomolar ranges [LogEC_50_ (α-MSH) = −8.938]. Wild-type MC4R had the constitutive activity, and the constitutive signal is thought to be responsible for the energy homeostasis disorder; several constitutive mutations of MC4R were found in obese people (20). The potential relevance of MC4R to constitutive signals in therapy remains to be further studied. Here, we found that wtMRAP2 increased the constitutive activity and enhanced the maximal response to α-MSH stimulation (**Figure 3A**). Variants II and V also exhibited dramatic increases in the constitutive activity of MC4R (**Figures 3C, F** **)**, while no significant change was observed on the constitutive activity of MC4R with the other variants (**Figures 3B, D, E, G** **)**. The maximal cAMP response was enhanced when co-transfected with variants II, IV, and VI (**Figures 3C, E, G** **)**, while variants I, III, and V inhibited the EC_100_ stimulation level of α-MSH compared to that of wtMRAP2 (**Figures 3B, D, F** **)**. Furthermore, the EC_50_ value was decreased in the presence of variants I–III, V, and VI, indicating the increased sensitivity of MC4R to α-MSH (**Tables 1** and **2**). The constitutive activity and maximum response of variants I (transmembrane region inversion) and III (C-terminal inversion) were both reduced.

**Table 1:** Summary of the pharmacological regulation of six melanocortin receptor accessory protein 2 (mMRAP2) variants on melanocortin-4 receptor (mMC4R) signaling.

Variants	Structural conformation	Constitutive activity	Plateau response	EC_50_ of α-MSH	IC_50_ of SHU9119	Surface translocation
WT	Wild type	**↑**	**→**	**↓**	**↓**	**↓**
I	TM inversion	**↑**	**↓**	**↓**	**↓**	**↓**
II	N-terminal inversion	**↑**	**→**	**↓**	**↓**	**↓**
III	C-terminal inversion	**→**	**↓**	**→**	**↑**	**↓**
IV	N- and C-terminal inversion	**↑**	**→**	**→**	**→**	**→**
V	N- and C-terminal transposition	**↑**	**↓**	**↓**	**↓**	**→**
VI	Replacement of the C-terminal by inverted N-terminal	**↑**	**→**	**→**	**→**	**→**

“**↑**” increased; “**↓**” decreased; “**→**” no significant effect.

WT, wild type; α-MSH, alpha melanocyte-stimulating hormone.

**Table 2:** Statistical analysis of **Figure 3**.

		1:0	1:3	1:6
LogEC_50_ (α-MSH)				
3A	MRAP2	−8.938	−9.81	−10.34
3B	I	−8.938	−9.709	−10.02
3C	II	−8.938	−10.11	−10.1
3D	III	−8.938	−9.121	−9.939
3E	IV	−8.938	−8.979	−8.982
3F	V	−8.938	−9.148	−9.901
3G	VI	−8.938	−9.226	−9.403
LogIC_50_ (SHU9119)				
3H	MRAP2	−9.185	−9.428	−9.664
3I	I	−9.185	−9.353	−9.255
3J	II	−9.185	−9.563	−9.715
3K	III	−9.185	−8.913	−8.81
3L	IV	−9.185	−8.942	−9.047
3M	V	−9.185	−9.023	−9.354
3N	VI	−9.185	−8.878	−8.987

MRAP2, melanocortin receptor accessory protein 2; α-MSH, alpha melanocyte-stimulating hormone.

SHU9119 works as a potent human MC3R and MC4R antagonist and a partial MC5R agonist (9). To investigate the antagonism of SHU9119 on MC4R signaling, we also checked the SHU9119-induced inhibition of MC4R signaling in the presence of wild-type MRAPs or MRAP2 variants (ratios of 1:0, 1:3, and 1:6) (**Figures 3H–N**). SHU9119 inhibited MC4R signaling, whereas wtMRAP2 attenuated this inhibitory effect (**Figure 3H**). Variant II exhibited a similar IC_50_ with wtMRAP2 and showed reduced constitutive activity and declined maximum inhibition level, the same as wtMRAP2 (**Table 1**). Variants I, III, IV, and V enhanced the SHU9119-induced inhibitory effect of MC4R and reached the same maximum level of inhibition as MC4R alone. The concentration–response curve of variant VI almost matched that of MC4R alone. Since variant VI was the only construct that was lacking the C-terminus, it further demonstrated the importance of the C-terminus in the regulation of MC4R signaling.

### Constitutive Activity of MC4R in the Presence of Six MRAP2 Variants

**Figure 4:**
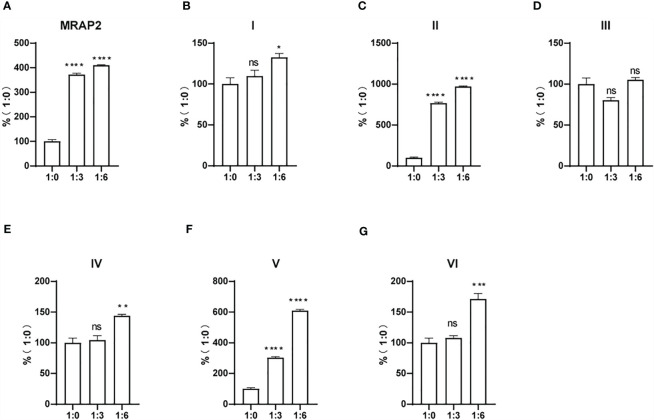
Pharmacological modulation of the constitutive activities of melanocortin-4 receptor (MC4R) by melanocortin receptor accessory protein 2 (MRAP2) variants. **(A–G)** cAMP production measurement of the constitutive activities of MC4R in the presence of various doses of the MRAP2 variants. Data are shown as the mean ± SEM and analyzed using two-tailed test. *ns* (no significant change), *p < 0.05,**p < 0.01, ***p < 0.001, ****p < 0.0001. Each *data column* represents the mean ± SEM of three replicates (*N* = 3).

As shown in **Figure 4**, the enhancement of the constitutive activity of MC4R weakened or even disappeared when co-transfected with variants I, III, and IV compared to that of wtMRAP2 and other variants, which indicated that the transmembrane domain and the C-terminus of MRAP2 played a vital role in modulating MC4R signaling and illustrated a fact that the internal orientation of the MRAP2 dimers is essential for sensitizing MC4R activation. In addition, we were also surprised to find that variants II and V could further improve the constitutive activity of MC4R compared with that of the wild type, especially variant II, which simultaneously increased the constitutive activity and maximum activity of MC4R. Accumulative results on the MRAP2 variants provided novel inspiration for the treatment of GPCR-associated disorders by optimizing the structural topology of accessory proteins in the future.

### Intracellular Translocation of MC4R in the Presence of MRAP2 Variants

**Figure 5:**
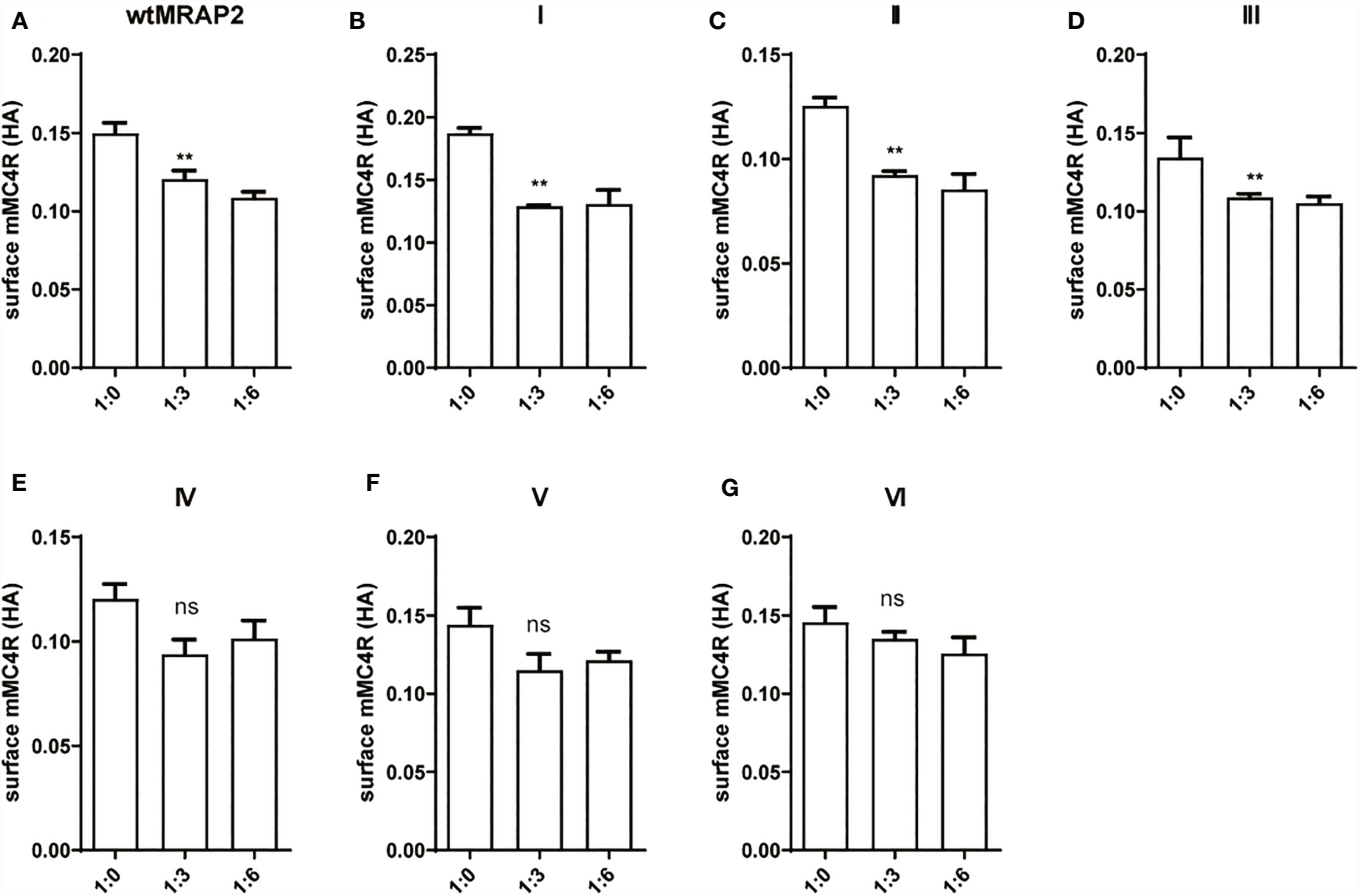
Surface trafficking measurement of melanocortin-4 receptor (MC4R) by melanocortin receptor accessory protein 2 (MRAP2) variants. Surface expression of MC4R was measured using ELISA in HEK293T cells transfected with 3HA-MC4R and different amounts of 2FLAG-MRAP2 or 2FLAG variants **(A–G)**. One-way ANOVA with *post-hoc* Tukey’s test. *ns* (no significant change), **p < 0.01. Each *data column* represents the mean ± SEM of three replicates (*N* = 3).

To determine whether the change of sensitivity of MC4R in the presence of the MRAP2 variants was related to the trafficking of MC4R to the cell surface, we measured the cell surface expression level of HA-tagged MC4R when co-expressed with certain ratios of wtMRAP2 or MRAP2 variant plasmids *via* enzyme linked immunosorbent assay (ELISA). As shown in **Figure 5**, the surface expression of MC4R was bidirectionally regulated by wtMRAP2 and the MRAP2 variants. The presence of wtMRAP2 and variants I, II, and III suppressed MC4R surface expression (**Figures 5A–D**). No statistically significant difference in MC4R surface expression was observed with the different amounts of MRAP2 IV, V, and VI (**Figures 5E–G**).

### The Co-Locolization of MC4R and MRAP2 Variants and Homodimer Formation of MRAP2 Variants on Plasma Membrance

**Figure 6:**
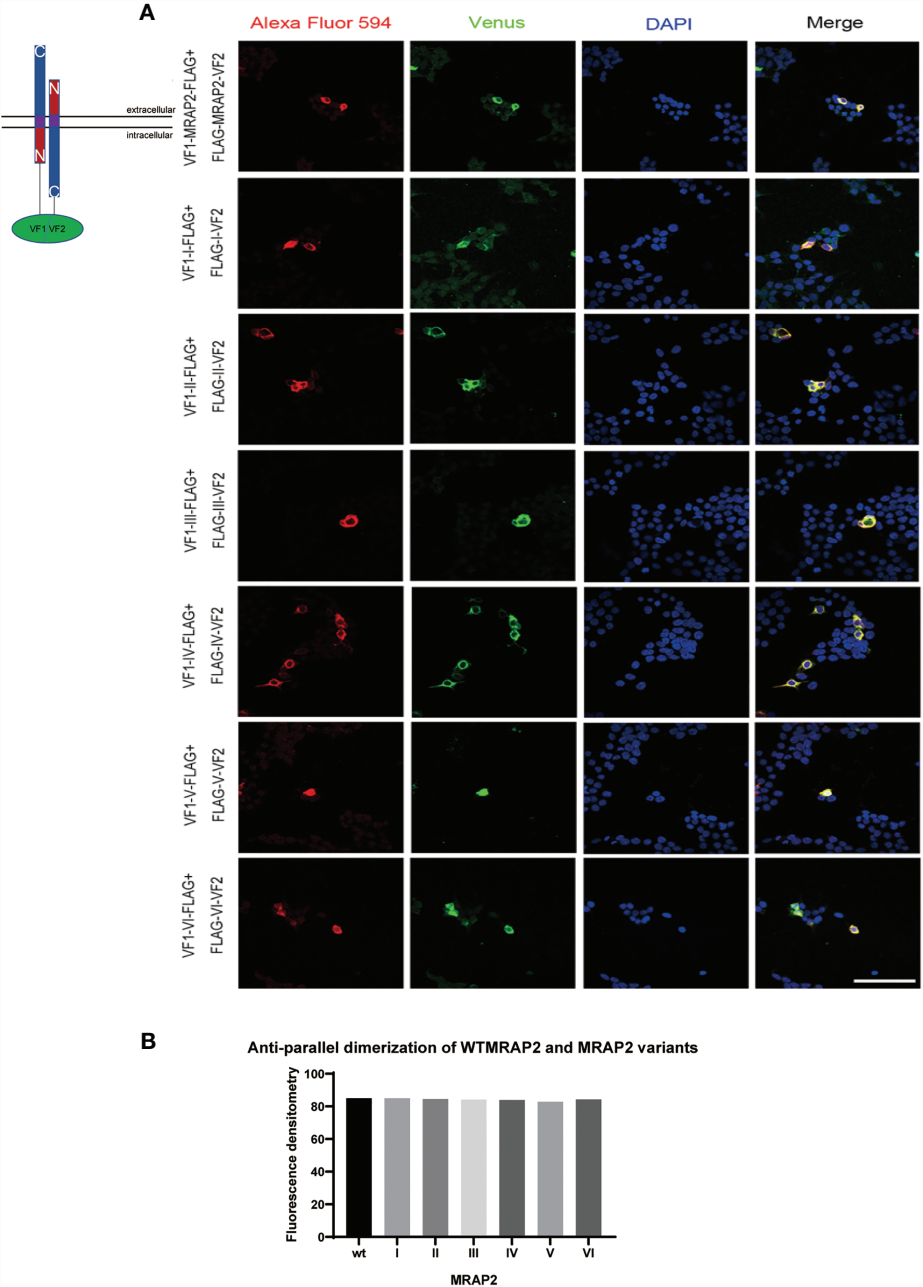
Antiparallel dimerization of melanocortin receptor accessory protein 2 (MRAP2) and the MRAP2 variants. **(A)** HEK293T cells transfected with plasmid constructs containing Venus fragments on opposite sides of MRAP2 or the MRAP2 variants. Alexa Fluor 594 (*red*) indicated the fluorescent secondary antibody. Venus fluorescence (*green*) indicated the MRAP2 antiparallel homodimers. Nuclei stained with DAPI are shown in *blue*. All three channels were merged in the *last photo of the panel*. *Scale bar*, 50 μm. **(B)** Fluorescence densitometry analysis on the images in **(A)**. Each *data column* represents the mean ± SEM of three replicates (*N* = 3).

**Figure 7:**
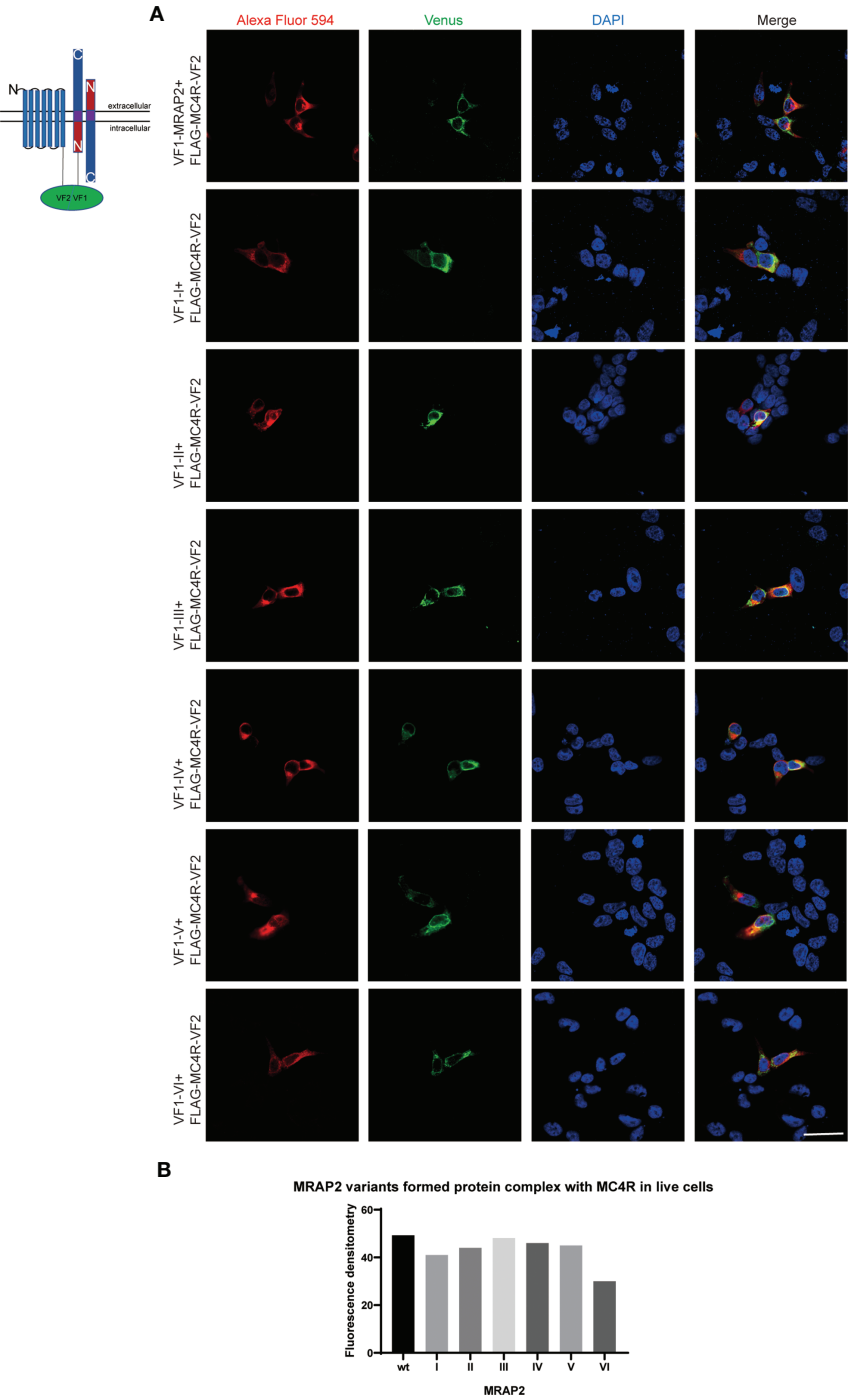
Melanocortin receptor accessory protein 2 (MRAP2) and all the variants formed protein complexes with melanocortin-4 receptor (MC4R). HEK293 cells were transfected with plasmids encoding VF1-X-FLAG and FLAG-MC4R-VF2. Alexa Fluor 594 (*red*) indicated the fluorescent secondary antibody. Venus fluorescence (*green*) indicated the MRAP2 antiparallel homodimers. Nuclei stained with DAPI are shown in *blue*. All three channels were merged in the *last photo of the panel*. *Scale bar*, 50 μm. **(B)** Fluorescence densitometry analysis on the images in **(A)**. Each *data column* represents the mean ± SEM of three replicates (*N* = 3).

MRAP2 has been reported to form the unique antiparallel homodimer in many species including mouse (21–24). To investigate whether the six mMRAP2 variants could still form the antiparallel homodimer, we performed the bimolecular fluorescence complementation (BiFC) assay. Venus fragments (VF1 and VF2) were respectively fused to the N- or C-terminal of MRAP2 and its variants. VF1-MRAP2 and MRAP2-VF2 were co-transfected in HEK293T cells. The results indicated that all variants could still form the proper antiparallel homodimer on the plasma membrane (**Figure 6**). Since MRAP2 and its variants could regulate the signaling of MC4R, we then performed BiFC assay to further check whether they could co-localize in live cells on the plasma membrane, as previously reported (25). The Venus fragments (VF1 and VF2) were respectively fused to the N- and C-terminals of MC4R and the MRAP2 variants. The green fluorescence could only be observed when the two fragments are very close to each other. Several studies have reported that MC4R locates at the cell membrane and co-localize with MRAP2 in live cells (23, 24, 26). Here, the steadily detectable fluorescence in **Figure 7** indicated the co-localization of MC4R and MRAP2 or its variants and varified the direct interaction and formation of tight functional protein complexes, as detected in **Figure 2**. There was no significant difference in the fluorescence density between the dimeric groups, as seen in **Figure 6B**. However, the fluorescence of the yellow fluorescent protein (YFP) between variant VI and MC4R showed a significant reduction compared with that of the other groups (**Figure 7B**). These data were also consistent with the co-IP results in **Figure 2G**.

## Discussion

MC4R plays an important role through MRAP2 in maintaining mammalian energy homeostasis. MC4R and MRAP2 knockout mice display metabolic disorders (27). Several MRAP2 variants reported from severe obese human patients affect MC4R signaling upon agonist stimulation (28). In particular, MRAP2 has a special membrane topology and is the only known eukaryotic protein that penetrates the membrane in both directions. Despite the central role of MRAP2 in energy balance, the orientational characteristics within MRAP2 that dominate membrane direction and dimerization are still unclear. Here, we focused on the pharmacological features of the intrinsic topology of MRAP2 and investigated the effect of different artificially constructed topological MRAP2 variants on MC4R signaling. To our knowledge, this is the first report on the internal symmetry of MRAP2. We reversed the amino acid sequence of each interior domain to investigate the function of each MRAP2 mutant (**Figure 1**). Like wtMRAP2, all variants could interact with itself, suggesting that the internal transposition did not affect the homodimerization of MRAP2 *in vitro* (**Figures 2A–F**). The replacement of the C-terminal with the inverted N-terminal could still form a dimeric topology, indicating that the essential motif required for dimer formation should be within the N-terminal region of mMRAP2. Furthermore, as shown in **Figure 6**, the immunofluorescence approach proved that the six variants could form the antiparallel homodimer on the cell surface. No matter how the amino acid sequence was inverted, it did not affect the formation of stable symmetrical dimers. We have previously shown the formation of antiparallel and parallel dimers of zebrafish MRAP2 (29). The sequence transposition did not result in the alteration of dimeric formation, which indicated that the mouse MRAP2 could form both antiparallel and parallel dimers as well. Additionally, the presence of a “doublet band” for MRAP2 variant V indicated that the N-terminal asparagine residue existed in both glycosylated and unglycosylated forms (**Figure 2E**). Co-IP was utilized to detect the interaction between MC4R and the MRAP2 variants (**Figure 2G**). As expected, we found that all MRAP2 variants interacted with MC4R, but with different intensities. Moreover, dopamine receptor D2, as a negative GPCR control, showed no interaction with all MRAP2 variants (**Figure 2G**). Like many other vertebrates, we confirmed that mMC4R was capable of interacting with wtmMRAP2 and all six variants *in vitro*, as shown by BiFC (**Figure 7**). We validated the fact that, although domain transposition resulted in a preference for interaction with MC4R, all variants still formed proper dual topology in live cells.

Dimerization or higher-order oligomers of MRAP2 may underlie the concentration-dependent effect of the regulatory action of MRAP2 on MC4R (16–19). Here, we showed that MC4R activation could be modulated by all MRAP2 variants by examining the potency of α-MSH and SHU9119 for MC4R in the presence of the MRAP2 variants (**Figure 3**). However, compared with the wtMRAP2, each variant showed different effects on the ligand sensitivity, constitutive activity, and maximum activity of MC4R. MC4R showed lower constitutive and maximum potential when co-expressed with variants I (transmembrane region inversion) and III (C-terminal inversion), which indicated that the transmembrane domain and the C-terminus of MRAP2 played a crucial part in modulating MC4R signaling. We also elucidated that the internal orientation of the MRAP2 dimers was important for sensitizing MC4R activity. It was reported that agonists and inverse agonists could affect the stability of receptors at the cell surface (30). The constitutive activity of melanocortin receptor was also partially related to its cell surface expression (31). The surface translocation of MC4R decreased when co-transfected with the MRAP2 variants (**Figure 4**). The differential tendency of the MC4R constitutive activity and its surface expression caused by the MRAP2 variants suggested that MRAP2 might also influence the conformation of MC4R in addition to its role in receptor surface translocation. In addition, with respect to the sensitivity measurement of α-MSH and SHU9119, it indicated that the induced changes in cell surface expression were not exclusively a result of the increased sensitivity. For example, the changes in energy balance could also affect the hypothalamic melanocortin receptor expression levels (32).

GPCR accessory proteins are small single-membrane proteins. Most accessory proteins are inserted into the membrane in one direction, such as Nexo/Ccyt (RAMPs) or Ncyt/Cexo (RTPs and REEP) orientation. MRAP2 differs from other accessory proteins in that it has an extremely unusual dual antiparallel topology (11). The inversion of the amino acid sequence did not affect the formation of mMRAP2 dimers, but it affected the binding of the mMRAP2 to mMC4R and the agonist α-MSH and also reduced the amount of mMC4R on the plasma membrane. The dual luciferase reporter assay demonstrated that the accessory proteins could regulate the sensitivity of mMC4R to its natural agonist. Collectively, the TM and the C-terminal of mMRAP2 were essential for modulating the constitutive activity of mMC4R, and the reciprocal orientation of the N- and C-terminals played a key role in regulating the sensitivity of mMC4R to natural and artificial ligands.

Here, our study investigated the internal symmetry of the functional symmetric antiparallel homodimer topology of MRAP2. Inversion of the N-terminal, transmembrane, or the C-terminal domain did not affect the dimeric formation, but resulted in the alteration of the regulatory role of MRAP2 dimers, the effect on MC4R trafficking, and the ligand-stimulated cAMP signaling of MC4R, suggesting the essential requirement of the proper orientation of functional motifs within the single-transmembrane accessory protein. Overall, this study is the first exploration examining the antiparallel homodimer structure of mMRAP2, and the alteration of the internal symmetry slightly changed their regulatory properties on mMC4R signaling. Essential motifs within the TM and the N- and C-terminal domains played individual and reciprocal roles in regulating the signaling cascades of multiple GPCR targets. Some of the variants appeared to be more efficient than did the wild type. For example, the variant I we created in this study helped MC4R to achieve both higher constitutive activity and maximum stimulatory activity, and variant V enhanced the constitutive activity of MC4R as well. These results provided us with a new inspiration to allosterically modulate GPCR activities by optimizing the structural topology of transmembrane accessory proteins, which could potentially lead to novel therapeutic applications to treat GPCR-associated disorders in the future.

## Methods and Materials

### Expression Constructs and Reagents

Wild-type MRAP2 and MC4R were amplified from complementary DNA (cDNA) library of mouse brain tissue. The original protein sequence of the variants was obtained by PCR cloning from wtMRAP2, and the inverted sequence was synthesized from GENEWIZ (South Plainfield, NJ, USA). Finally, primers were designed to assemble the entire variant sequences. All plasmids were verified by DNA sequencing. 2FLAG-tagged/3HA-tagged wtMRAP2 and MRAP2 mutants, 3HA-tagged MC4R, 3HA-tagged D2dr, 2FLAG-tagged RAMP3, VF1-MRAP2, FLAG-MRAP2-VF2, and FLAG-MC4R-VF2 expression constructs were subcloned into pcDNA3.1 vectors. The pCre-luc (Santa Cruz Biotechnology, Santa Cruz, CA, USA) and pRL-TK (Promega, Madison, WI, USA) plasmids were kind gifts from Xin Xie’s Lab of Tongji University. α-MSH was obtained from Genescript (Nanjing, China). 3,3′,5,5′-Tetramethylbenzidine (TMB) substrate solution was purchased from Beyotime® Biotechnology (Shanghai, China). Mouse monoclonal anti-HA (Sigma-Aldrich, MO, USA), anti-FLAG (ABclonal Biotech Co., Ltd, Wuhan, China), and horseradish peroxidase (HRP)-conjugated antibodies against mouse (ABclonal Biotech Co., Ltd, Wuhan, China) were used in this study. The primers used in this study were all synthesized from GENEWIZ.

### Cell Culture and Transfection

HEK293T cells were cultured in Dulbecco’s modified Eagle’s medium (DMEM) supplemented with 10% (*v*/*v*) fetal bovine serum (FBS) and 1% penicillin/streptomycin. Cells were incubated in a humidified atmosphere consisting of 5% CO_2_ at 37°C. PEI (Polyethyleneimine) transfection reagent was used to perform transfection at 70%–90% confluency according to the manufacturer’s protocols. During transfection, the total amounts of plasmid were kept constant for all groups by adding empty pcDNA3.1(+) vector. All experiments were conducted 24 h post-transfection.

### Western Blot and Co-Immunoprecipitation

HEK293T cells were co-transfected with N-terminally 3HA-tagged and N-terminally FLAG-tagged MRAP2 wild-type (WT) or mutant constructs using PEI following the manufacturer’s instructions. At 24 h post-transfection, the cells were washed with ice-cold PBS and lysed with Western and IP cell lysis buffer (P0013, Beyotime) containing proteinase inhibitor cocktail (Roche, Penzberg, Germany). The lysed cells were rotated for 1 h at 4°C and then were collected at 17,000 × *g* at 4°C for 15. Then, 10% lysate added with 5× protein loading buffer (C508320-0010, Sangon Biotech, Shanghai, China) was frozen at −20°C as the total lysate sample; the remaining supernatants were used for co-IP by incubation with the indicated antibody (mouse anti-HA or mouse anti-FLAG) at 1:5,000 dilution overnight at 4°C with rotation. Protein A+G agarose beads (P2055, Beyotime) were added to the IP tubes the next day and the mixture was rotated for 4 h at 4°C. The beads were washed three times with lysis buffer. The proteins from the beads were eluted with 5× protein loading buffer. Both total lysate and IP samples were boiled at 95°C for 15 min. The eluted proteins and whole cell lysates were separated using SDS-PAGE on a 12% gradient gel (BBI, C661102, Shanghai, China) and transferred to polyvinylidene fluoride membranes. The membranes were blocked for 15 min with QuickBlock Western block buffer(P0252, Beyotime) at room temperature. Either mouse FLAG or mouse HA primary antibodies (Abcam, Cambridge, UK) were used at a 1:5,000 dilution in QuickBlock Primary Antibody Dilution Buffer for Western Blot (P0256, Beyotime) overnight at 4°C The blots were washed three times for 5 min with Tris-buffered saline–Tween 20 (TBST). A secondary rabbit anti-mouse HRP-conjugated antibody (Abcam) was used at a 1:5,000 dilution for 2 h at room temperature. The blots were washed (three times for 5 min) with TBST before adding the BeyoECL Star Western blotting substrate (P0018, Beyotime). The blots were imaged using the Amersham Imager 600 System.

### cAMP Luminescent Assay

HEK293T cells were seeded into 24-well plates and cultured to reach about 70% confluency. The MC4R plasmid was co-transfected with wtMRAP2 or each MRAP2 variant construct at ratios of 1:0, 1:3, and 1:6 (empty vectors were added to each group to achieve the exact same transfection quantification), along with the pCRE-Luc and pRL-TK (Renilla luciferase) reporter vectors into HEK293 cells per well *via* PEI reagent. After 24 h transfection, the medium was replaced with 0.1% bovine serum albumin (BSA) in DMEM supplemented with different concentrations of α-MSH and incubated for 4 h at 37°C, 5% CO_2_. Renilla luciferase was used as an internal control and was transfected into cells in order to avoid errors caused by differences in the transfection efficiency. The cAMP level was measured using the Dual-Glo Luciferase Assay System (Promega) as described by the manufacturer. Briefly, for the first use, we prepared LAR II, the substrate of Firefly luciferase. LAR II was dissolved in LAR II buffer and stored in aliquots at −80°C and protected from light. After α-MSH incubation, the growth medium was removed from cultured cells, 1× PLB was added, and the cells were lysated at room temperature for 15 min. The lysates were transferred into a new, white 96-well plate and the fluorescence value determined (Firefly luciferase) with a Spectramax M5 plate reader. Subsequently, the Stop&Glo buffer (the substrate of Renilla luciferase) was added to stop the LAR II reaction and was read again. For data processing, we first calculated the ratio of Firefly luciferase/Renilla luciferase per group and then used the ratio of the control group (without α-MSH treatment) as unit 1 in order to obtain the relative luciferase activity of the different treatment groups. Finally, we obtained the regulation activity of MC4R in each group.

### Cell Surface ELISA

Twenty-four-well plates were pretreated with poly-l-lysine solution and the HEK293T cells were seeded. Then, the cells were transiently transfected with the indicated plasmids. At 24 h post-transfection, the cells were washed with PBS three times and fixed for 20 min with 4% paraformaldehyde at room temperature. The cells were then blocked with 5% milk in PBS for 30 min. Next, the samples were incubated with 1:4,000 mouse anti-HA antibodies (Abcam) for 2 h and incubated with 1:5,000 HRP-conjugated secondary antibodies for 1 h at room temperature. Finally, the samples were incubated with TMB for 10–20 min under dark, the reaction was terminated with 2 M sulfuric acid, and the mixture was transferred into a new transparent 96-well plate as the surface expression test group. The cells remaining on solid surface (typically in 24-well plates) were stained by adding 1X Janus Green stain. The cells were incubated for 5 min at room temperature. The dye was then removed and washed five times in ultrapure water. The last wash was removed, 0.1 ml 0.5 M HCl was added into each well, and incubated for 10 min. After shaking the plate for 10 s, the mixture was transferred into a new 96-well transparent plate as the Janus Green test group. The OD (optical density) value was recorded at 450 nm; Janus Green OD was measured at 595 nm on a Spectramax iD3 multimode plate reader.

### Immunofluorescence

HEK293T cells were seeded into 12-well plates with slides previously coated with poly-d-lysine. The cells were transfected with VF1-X and FLAG-X-VENUS-F2, or VF1-X and FLAG-MC4R-VENUS-F2 plasimds the following day. At 24 h post-transfection, the cells were washed with PBS three times and fixed with 4% paraformaldehyde for 20 min. Then, the cells were again washed with PBS (three times for 5 min). They were then blocked with 5% milk in PBS for 30–40 min. Blocked cells were then incubated with a mouse FLAG antibody (Abcam) at a 1:1,000 dilution in 5% milk in PBS overnight at 4°C. The next day, the cells were washed with TBST three times and were incubated with a rabbit anti-mouse Alexa 594 antibody (Abcam) at a 1:1,000 dilution in 2% BSA in PBS for 1 h in the dark. The cells were washed with TBST (three times for 5 min) and the slides mounted withProLong(R) Gold Antifade with DAPI Molecular Probes (8961S, Cell Signaling, Danvers, MA, USA). Images were captured with a Zeiss confocal microscope and analyzed by ImageJ software.

### Data Analysis

All experiments in this study were conducted in triplicate and repeated separately at least three times. Statistical differences were analyzed with GraphPad Prism6 (GraphPad software). One-way ANOVA with Tukey’s *post-hoc* test was used to determine the statistically significant differences between the control and the experimental groups. The tests were performed at nominal significance levels of ns (no significant change), *p < 0.05, **p < 0.01, ***p < 0.001, ****p < 0.0001.

## Data Availability Statement

The original contributions presented in the study are included in the article/supplementary material. Further inquiries can be directed to the corresponding authors.

## Author Contributions

MW, LP, LeL, and ChZ participated in the design of the study. MW, XL, and LP performed the experiments. JX, ZK, and CoZ contributed to data collection and data analysis. MW, LP, and ChZ contributed to the writing of the manuscript. All authors agree with the order of presentation of the authors. All authors contributed to the article and approved the submitted version.

## Funding

The work was supported by grants from the National Key Research and Development Program of China (grant nos. 2017YFA0103902 and 2019YFA0111400), the National Natural Science Foundation of China (grant no. 31771283), Shanghai Municipal Key Clinical Specialty (Grant No. shslczdzk00901), the Fundamental Research Funds for the Central Universities of Tongji University (no. 22120190210), Innovative Research Team of High-Level Local Universities in Shanghai (grant no. SSMU-ZDCX20180700), and the Key Laboratory Program of the Education Commission of Shanghai Municipality (no. ZDSYS14005).

## Conflict of Interest

The authors declare that the research was conducted in the absence of any commercial or financial relationships that could be construed as a potential conflict of interest.

## Publisher’s Note

All claims expressed in this article are solely those of the authors and do not necessarily represent those of their affiliated organizations, or those of the publisher, the editors and the reviewers. Any product that may be evaluated in this article, or claim that may be made by its manufacturer, is not guaranteed or endorsed by the publisher.
